# Identification of NQO2 as a protein target in small molecule modulation of hepatocellular function

**DOI:** 10.1021/acschembio.1c00503

**Published:** 2021-08-24

**Authors:** Arnout G. Schepers, Jing Shan, Andrew G. Cox, Ada Huang, Helen Evans, Chad Walesky, Heather E. Fleming, Wolfram Goessling, Sangeeta N. Bhatia

**Affiliations:** 1 Koch Institute for Integrative Cancer Research, Massachusetts Institute of Technology, Cambridge, MA 02139, USA; 2 Netherlands Cancer Institute, Amsterdam, Netherlands; 3 Genetics Division, Brigham and Women’s Hospital, Harvard Medical School, Boston, Massachusetts, USA; 4 Peter MacCallum Cancer Centre, Melbourne, Victoria, Australia; 5 Harvard Stem Cell Institute, Cambridge, Massachusetts, USA; 6 Dana-Farber Cancer Institute, Boston, Massachusetts, USA; 7 Division of Gastroenterology, Massachusetts General Hospital, Boston, Massachusetts, USA; 8 Harvard-MIT Division of Health Sciences and Technology, Institute for Medical Engineering and Science, Massachusetts Institute of Technology, Cambridge, MA 02139, USA; 9 Broad Institute of Massachusetts Institute of Technology and Harvard, Cambridge, MA 02139, USA; 10 Electrical Engineering and Computer Science, Massachusetts Institute of Technology, Cambridge, MA 02139, USA; 11 Department of Medicine, Brigham and Women’s Hospital and Harvard Medical School, Boston, MA 02115, USA; 12 Howard Hughes Medical Institute, Cambridge, MA 02139, USA; 13 Wyss Institute at Harvard, Boston, MA 02115, USA

**Keywords:** Small molecule inhibition, FH1, in vitro liver models, zebrafish liver model, target identification, NQO2, liver functionality

## Abstract

The utility of in vitro human disease models is mainly dependent on the availability and functional maturity of tissue-specific cell types. We have previously screened for and identified small molecules that can enhance hepatocyte function in vitro. Here, we characterize the functional effects of one of the hits, FH1, on primary human hepatocytes in vitro, and also in vivo on primary hepatocytes in a zebrafish model. Furthermore, we conducted an analogue screen to establish the structure-activity relationship of FH1. We performed affinity-purification proteomics that identified NQO2 to be a potential binding target for this small molecule, revealing a possible link between inflammatory signaling and hepatocellular function in zebrafish and human hepatocyte model systems.

## Introduction

In vitro models of human tissue can facilitate the study of human diseases and assist in the development of safe and effective drug therapies. The liver is the largest gland in the body, and performs diverse metabolic, secretory, and inflammatory response roles, which makes it a challenging organ to model in vitro. Existing tools used to assess the risk of hepatotoxicity and/or to study the pathology of liver disease include animal models, as well as in vitro platforms that incorporate hepatocytes, either alone or in co-culture with other cell types, both in 2D and 3D formats ^1–3^. Recently, 3D models have also been integrated with microfluidics to dynamically mimic organ functionality ^4^. Most in vitro models are based on primary human hepatocytes (PHHs), which constitute about two thirds of the total cell population in the healthy liver parenchyma. However, when maintained under an artificial physicochemical environment, PHHs progressively lose hepatocyte-specific functions and morphology ^5^. The decline in viability and liver-specific functions *in vitro* is thought to be caused in part by disruption of the normal tissue architecture, as well as due to adaptation to the in vitro environment. During the isolation process from the donor liver, cell-cell connections and cell-matrix interactions are disrupted. In addition, ischemia-reperfusion injury induces activation of intracellular inflammatory pathways ^6^. The phenotype of isolated PHHs can be partially stabilized through co-culture with both liver- and nonliver-derived nonparenchymal cell types ^7–9^. In addition, cell-derived matrices have been used to mimic the liver microenvironment and uphold a subset of liver-specific functions including albumin synthesis, urea production, and P450 activity, though these systems still suffer from progressive loss of functionality ^5,10^. The use of cocktails of small molecules has been reported to maintain the mature function of cultured PHHs ^11^, and understanding the underlying mechanisms triggered by these drug stimuli may provide new opportunities to create more accurate in vitro disease models.

We have previously developed a high-throughput screening platform to identify small molecules that can either induce proliferation of mature PHHs or promote maturation of human iPS-derived hepatocyte-like cells ^12^. The most potent functional hit from that screen, FH1, can also be used to boost in vitro functions of hepatocytes. Here, we further characterized the effects of FH1 and identify a binding target to better understand the pathways involved. Gene expression profiling of PHHs upon FH1 treatment revealed altered TNFα, IL-6, and STAT3 signaling; all major regulators of inflammation and regeneration. In vivo, FH1 treatment enhanced liver size during zebrafish development and improved survival following lethal doses of acetaminophen, in embryos and as well as in adult animals. Using affinity pull-down mass spectrometry, we identified ribosyldihydronicotinamide dehydrogenase (NQO2) as a potential target of FH1. We demonstrate that FH1 binds to and inhibits the function of NQO2, and that knockdown of NQO2 can enhance the albumin secretion, a central hepatocyte function, of our PHH-containing liver cultures.

## Results

### FH1 increases functionality of in vitro liver co-cultures.

**Figure 1.**
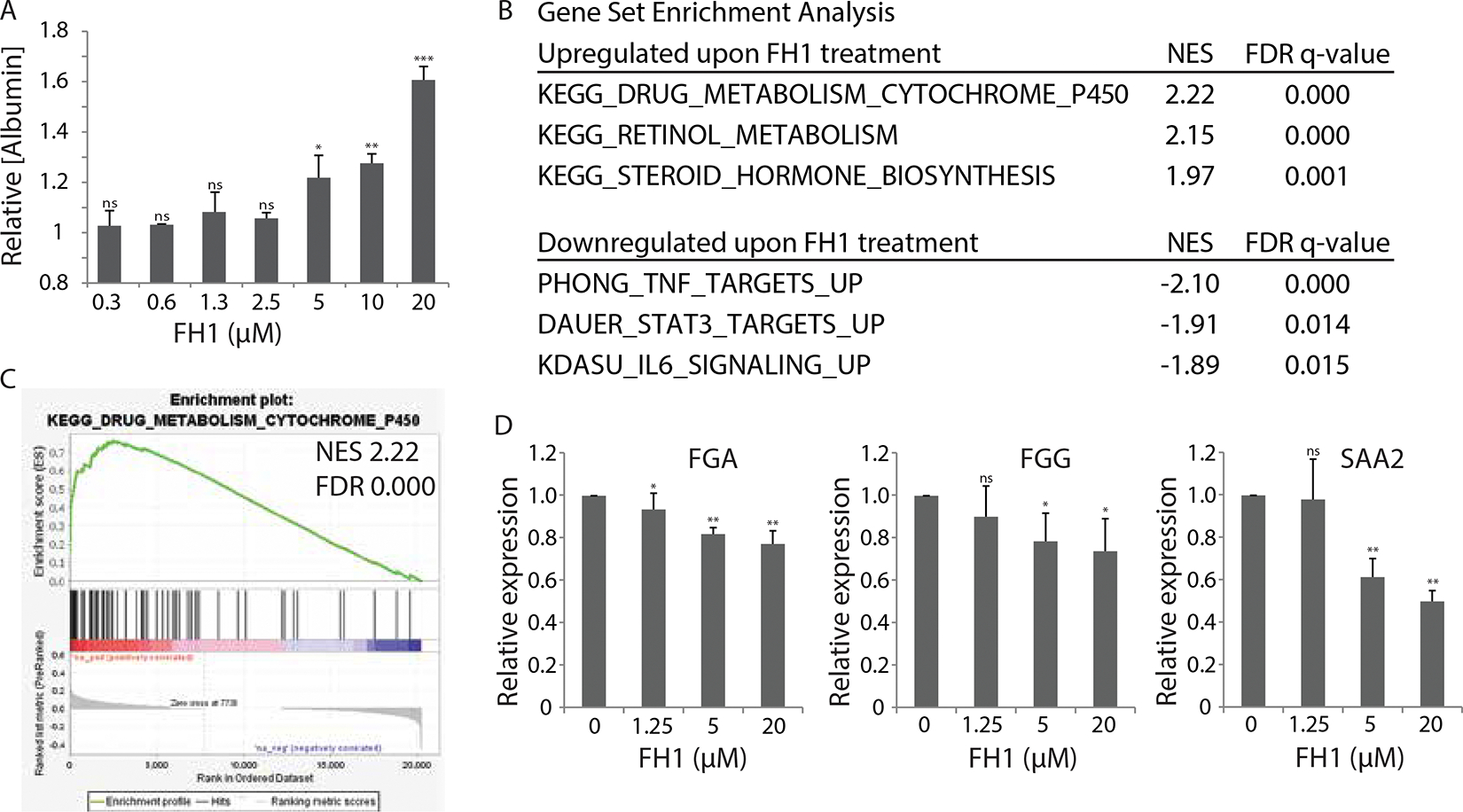
FH1 increases functionality of in vitro liver co-cultures A) Relative albumin secretion of co-cultures of PHHs and 3T3-J2s after 48 hours of treatment with increasing doses of FH1, normalized to DMSO-treated cultures. Error bars represent SEM for biological triplicates. B) Overview of Gene Set Enrichment Analysis shows enrichment of genes involved in drug metabolism and other metabolic pathways, and reduced expression of genes involved in TNFa, IL-6, and STAT3 signaling upon treatment of FH1. C) Gene set enrichment analysis of altered genes in co-cultures treated for 24 hours with 20μM of FH1 vs DMSO shows enrichment of genes involved in drug metabolism by cytochrome P450. (NES = Normalized enrichment score, FDR = False discovery rate). D) Dose-dependent upregulation of mRNA expression of mature liver function genes upon treatment with FH1 for 24 hours. Graphs show average results (Fragments per Kilobase Million) normalized to DMSO for FGA, FGG and SAA2. Error bars are SEM and represent the variation between three different hepatocyte donors. Asterisks represent P-values for the difference between treated samples and untreated controls (t-test; **=<0.01, ***=<0.001, ns=nonsignificant).

The small molecule FH1 was identified in a high-throughput screen of small molecules as a compound that can improve the secretion of albumin by mature PHHs, as well as promote the differentiation of iPS cells toward a hepatic lineage^12^. Consistent with our previous findings, when we added FH1 to PHHs that were co-cultured on a mouse fibroblast (3T3-J2) feeder layer, we observed a dose-dependent increase in human albumin protein secretion (Fig. 1a), traditionally regarded as a biomarker for synthetic function of hepatocytes^13^. The same positive trend was observed with PHHs cultured in a monolayer on collagen-coated plates, without supporting fibroblasts (supplemental figure S1), indicating that FH1 has the capacity to impact the hepatocytes directly. To explore possible underlying mechanisms responsible for FH1’s effect on hepatocytes, we treated co-cultures of PHHs and supportive fibroblasts with varying doses of FH1 for 24 hours, and analyzed gene expression by RNAseq. Since DMSO can have effects on the differentiation of hepatic cells^14^, the DMSO concentration in media with and without FH1 was controlled for and never exceeded 0.1%. Gene set enrichment analysis comparing exposure to 20μM FH1 versus DMSO control showed significant enrichment of transcripts involved in drug metabolism, retinol metabolism, and steroid hormone biosynthesis, consistent with a population that exhibited more robust liver function (Fig. 1b–c). Furthermore, FH1-treated cells expressed fewer transcripts of genes involved in fibrosis and liver inflammation, i.e. Nicotinamide N-Methyltransferase, Fibrinogen alpha, and Serum Amyloid A proteins (Table 1). Therefore, we compared our data to gene set signatures that correspond with inflammatory pathways, such as TNFα, IL6, and STAT3, and found significantly lower representation of these mRNAs in PHHs treated with FH1 (Fig. 1b). Comparing the results from PHHs derived from 3 independent donor liver sources treated with FH1 indeed shows a dose-dependent decrease of human-specific transcripts for inflammatory genes, e.g. Figure 1d shows the average relative mRNA expression values for FGA, FGG, and SAA2, where the error bars represent the variation between the three hepatocyte sources. Even though the gene set enrichment only captures a subset of liver functions, and does not take into account posttranslational protein modifications, these data point to the capacity of the small molecule, FH1, to support the function of hepatocytes, at least in part by reducing pro-inflammatory and fibrotic pathways.

### FH1 treatment protects against acetaminophen induced liver toxicity in vivo

**Figure 2.**
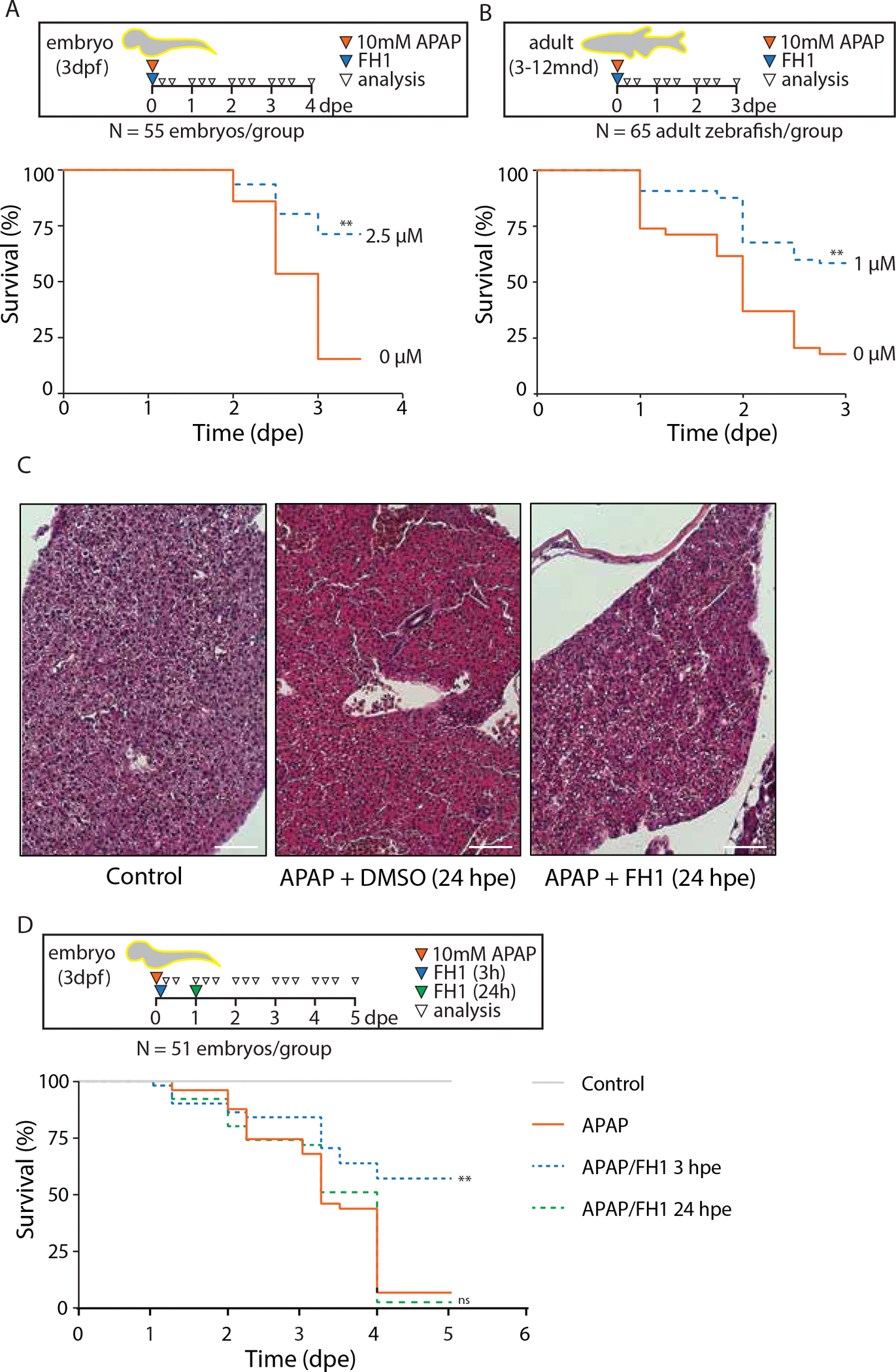
FH1 promotes fetal liver expansion and prevents lethal toxicity in zebrafish A) Three-day old embryos were treated with a lethal dose of acetaminophen (APAP) in combination with either FH1 or DMSO. The percentage of live animals was scored over time (dpe = days post exposure). B) 3–12 month old zebrafish were treated with a lethal dose of APAP in combination with either FH1 or DMSO. C) Hematoxylin and eosin stain of liver tissue of adult zebrafish, 24 hours post exposure. Representative images of untreated controls and images following a lethal dose of APAP in combination with DMSO or FH1. Scale bars represent 50 μm. D) Three-day old embryos were treated with APAP, and FH1 was added either 3h or 24h after APAP exposure (For A, B and D; Log-rank Mantel-Cox test compared to APAP treatment alone; **=<0.01, ns=nonsignificant).

Although our liver co-cultures recapitulate several mature functions, they lack physiological complexity. As FH1 may affect inflammatory pathways that are regulated by the interplay of several cell types and systemic factors, we set out to test the effects of FH1 in vivo using a zebrafish model. The zebrafish liver contains the same main cell types that are found in human liver and exhibits similar pathogenic responses to environmental insults and genetic mutations ^15^. It is therefore a popular model for drug toxicology studies. Having observed that FH1 can reduce inflammation and support the function of hepatocytes in vitro, we sought to assay whether FH1 would extend a protective effect in response to liver damage. Acetaminophen overdose is one of the most common causes of drug-induced liver toxicity. Two factors in acetaminophen toxicity are formation of a reactive metabolite *N*-acetyl-*p*-benzoquinone imine (NAPQI) and production of reactive oxygen species (ROS) ^16^. The toxic effects can be recapitulated in a zebrafish model; acetaminophen depletes glutathione stores, elevates aminotransferase levels, increases apoptosis, and causes drug-dependent hepatocyte necrosis and death ^17^. Following this established pattern, after 3 days of exposure to 10mM acetaminophen, a mere 15% of embryos survived. Treatment with FH1 during acetaminophen administration led to a significant increase in survival of up to 71% (Fig. 2a). This hepatoprotective effect elicited by FH1 was also observed in developmentally mature, adult animals, where survival after acetaminophen treatment increased from 18% to 58% (Fig. 2b). Hematoxylin and eosin (H&E) staining of adult liver tissue showed more intense red staining 24 hours post exposure to acetaminophen, indicative of increased levels of denatured protein which bind the red dye, eosin. Denatured proteins either coagulate, resulting in necrosis, or bind heat shock proteins to trigger apoptosis. In contrast, livers treated with FH1 in addition to acetaminophen showed a reversal of this phenotype and resembled the control sections (Fig. 2c). Notably, we found that acetaminophen-treated embryos could be rescued when FH1 was administered 3h after exposure to the hepatotoxin. However, if the addition of FH1 was delayed till 24h after acetaminophen exposure, no survival improvement was observed (Fig. 2d). A similar time window for rescue is also observed with N-acetylcysteine (NAC), the standard treatment for acetaminophen toxicity, which protects the liver by replenishing glutathione stores and by inhibiting pro-inflammatory cytokines ^17,18^.

### FH1 target identification

Based on the patterns of maturation and hepatoprotection, and the gene set enrichment analysis after treatment with FH1, we hypothesized that FH1 may affect regulatory pathways in inflammation and regeneration processes. In order to determine how FH1 might mediate these effects, we sought to identify its direct binding partners by affinity-based proteomics.

**Figure 3.**
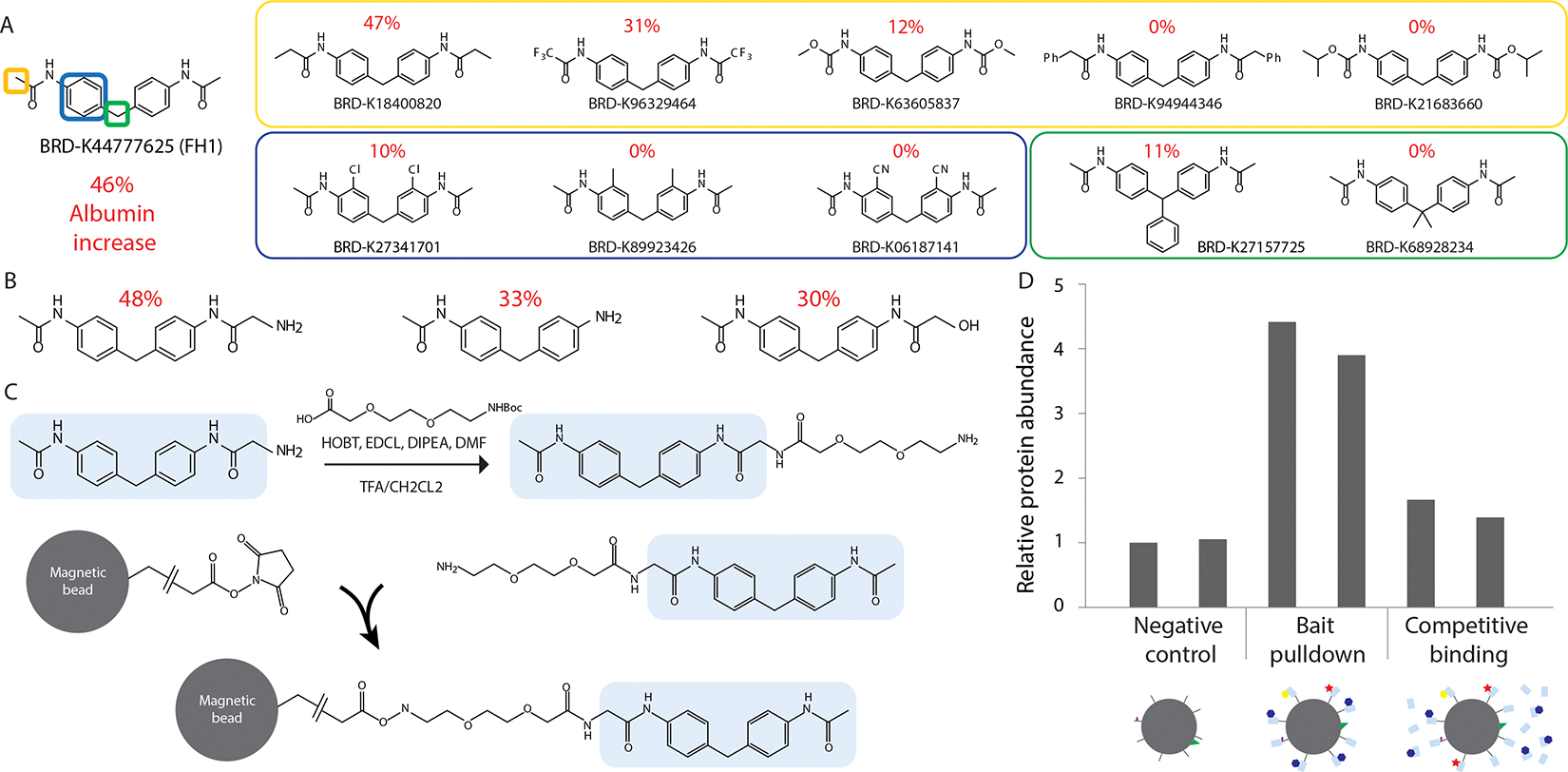
Target identification A) Analysis of structure-activity-relationship. Functional screen of analogues of FH1 with alterations at the edges (yellow), ring structure (blue), or the connection between the rings (green). Analogue molecules were examined for retention of the capacity to enhance liver co-culture functionality. The percentage in red indicates the increase in albumin secretion after modified small molecule treatment, compared to DMSO control. B) An additional functional screen of 24 custom analogues (Fig. S3) yielded three analogues, with reactive side groups that can accommodate additional binding partners (i.e. magnetic beads), that maintained the function of the original FH1 molecule. C) Affinity bait generation. Top reaction; schematic representation of double PEG linker addition and Boc deprotection. Bottom reactions: the small molecule analogue with linker was then coupled to NHS activated magnetic beads. D) Affinity pulldown coupled to mass spectrometry was done using lysates of freshly-thawed PHHs, in two biological replicates per condition. For the competitive binding control, lysates were pre-incubated with 100 μM FH1 small molecule for three hours prior to the addition of bait-beads. Relative abundance of peptides corresponding to NQO2 was enriched in the bait pulldown compared to the negative control and compared to the competitive binding control.

The initial, and perhaps most crucial, step to achieve a successful target identification by affinity pulldown is the generation of a purification-ready bait molecule. Structural modifications made in order to enable bead linkage commonly result in altered functionality. Accordingly, the structure-activity relationship needs to be determined for the target compound. Thus, we tested the function of a panel of 14 commercially-available analogues of FH1 under the same conditions as the original albumin secretion screen of PHH-fibroblast co-cultures^12^. When the analogues were grouped into subcategories of chemical variations, we found that a comparable increase in albumin secretion was maintained when a small side group was added to the edges of the FH1, whereas changing the ring structures or the connection between the rings led to highly disrupted function of the original molecule (Fig. 3a). With this information in hand, we synthesized 72 custom analogues with chemically-reactive side groups that would be amenable to the downstream chemistry needed to attach magnetic beads (Fig. S3) and identified three molecules that retained hepatocyte-supporting functionality (Fig. 3b).

A small molecule that is directly conjugated to a bead may not protrude far enough from the bead’s surface to permit its interaction with protein targets ^19^. To overcome this potential hurdle, we added an amino-PEG2 linker to the FH1 analogue with the highest hepatocyte-supporting functionality and bound the product to NHS-activated magnetic beads (Fig. 3c). Using these bait-beads, we performed affinity purification by incubating with lysates from freshly-thawed PHHs, followed by mass spectrometry to identify any proteins bound to the target beads, relative to material bound to control beads that included no small molecule or linker. Previous affinity purification studies have found that while stringent washing steps should release any non-specifically bound sample components, distinguishing true interaction partners from non-specific binders remains a significant challenge in the application of this procedure ^20,21^. Therefore, to complement the affinity-based purification process we also included a competitive binding control by pre-incubating lysates with free FH1 small molecule, prior to the addition of bait-beads ^22^. Using this strategy we observed a significant enrichment of Quinone Oxidoreductase 2 (NQO2) in the material that was bound to the FH1-complexed bait-beads, compared to the control beads. This enrichment was not observed in the bead material after pre-incubation of the lysate with free FH1 (Fig. 3d). Additional proteins were picked up in the pulldown process with affinity beads versus control beads, yet these enriched candidates did not exhibit a specific depletion after competitive pre-binding with free FH1 (Fig. S4). Furthermore, the FH1 molecule resembles two acetaminophen molecules linked together, and acetaminophen has been shown to bind to NQO2, ^23^ as well.

### FH1 binds and inhibits activity of NQO2

**Figure 4.**
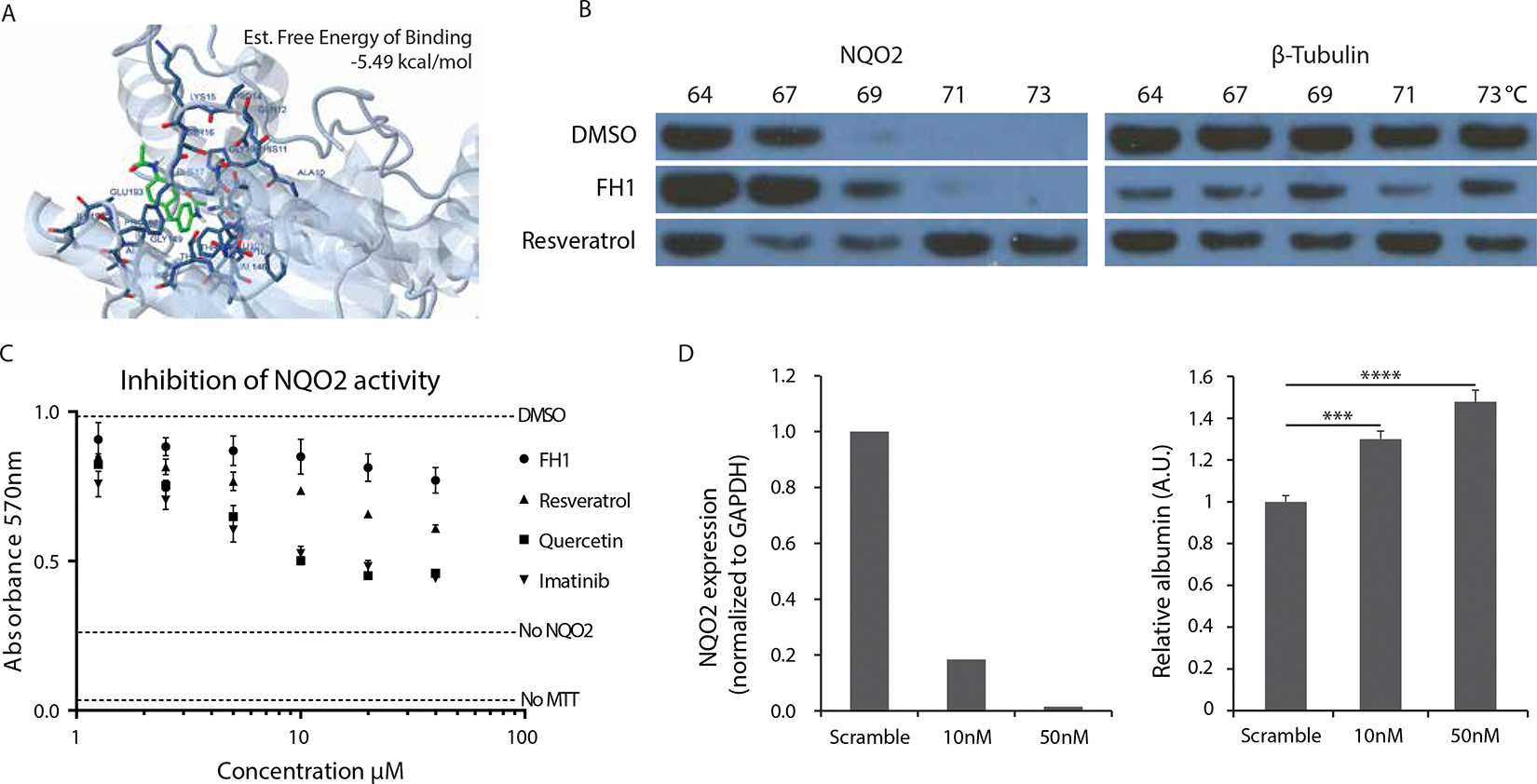
FH1 binds NQO2 and inhibits its function A) In silico docking of FH1 to NQO2. A schematic of the structure of NQO2, with a prediction of the conformation of its active site binding to an interacting FH1 molecule (depicted in green). Estimated free energy of binding was calculated with dockingserver. B) Cellular thermal shift assay (CETSA); PHHs were incubated for 24h with DMSO, FH1, or resveratrol. After 24h, the hepatocytes were trypsinized and cell suspensions were heated at the indicated temperature for 3 min and lysed. Cell lysates were centrifuged to pellet denatured protein, and proteins in suspension were analyzed by western blot to determine the relative preservation of NQO2. β-Tubulin is shown as a loading control. C) In vitro inhibition of NQO2 activity. Recombinant NQO2 was incubated with substrate in combination with increasing concentrations of potential inhibitors of NQO2; FH1, resveratrol, quercetin, and imatinib, and assayed for maintenance of NQO2 enzymatic activity. Dotted lines; ‘no MTT’ indicates the no substrate control, ‘no NQO2’ indicates the no enzyme control, ‘DMSO’ indicates the no inhibitor control. Assays performed in triplicate. Significance of inhibitory effect of each compound was assessed with a t-test of log-dose versus response (p<0.001 for each compound at a dose >2.5μM). D) Left: siRNA mediated knockdown of NQO2 expression in PHHs cultured on collagen-coated plates at two different concentrations of siRNA. Knockdown efficiency was determined 24h after transfection of siRNAs. Right: Knockdown of NQO2 results in higher levels of albumin secretion in PHHs, as measured by albumin ELISA 48h after addition of siRNA. Error bars represent SEM.

NQO2 is an FAD-containing phase II detoxification enzyme that uses dihydronicotinamide riboside (NRH) rather than NAD(P)H as an electron donor. Loss of NQO2 leads to altered intracellular redox status, decreased expression and activity of NF-κB and altered chemokines ^24^. NQO2 can also be bound by resveratrol ^25^, which has been shown to exhibit anti-inflammatory activity ^26^. To explore the interaction between FH1 and NQO2, we first performed in silico docking analysis using DockingServer ^27^. Since resveratrol has been established as a binding partner of NQO2 ^25^, we based our analysis on the crystal structure of NQO2 in complex with resveratrol. FH1 is predicted to bind to the active site of NQO2 with an estimated free energy of −5.49 kcal/mol (Fig. 4a), comparable to the estimated free energy for resveratrol (−5.72 kcal/mol, Fig. S5).

Binding of a small molecular weight ligand can increase the thermal stability of a protein ^28^. The change in thermal denaturation temperature can be quantified with a cellular thermal shift assay (CETSA), such that at increasing temperatures, a target protein will not denature and aggregate if stabilized by the binding of an appropriate ligand, and will therefore remain in sample solution upon centrifugation. Using CETSA in combination with western blot, we analyzed the stability of NQO2 after FH1 treatment. PHHs were incubated with DMSO, FH1, or resveratrol. After 24h incubation, the hepatocytes were trypsinized, and cell suspensions were heated at the indicated temperature for 3 min and then lysed. Cell lysates were centrifuged to remove the denatured protein, and proteins in suspension were analyzed by western blot to determine the relative preservation of NQO2. The results show that FH1 increased the thermal stability of NQO2, although not to the same extent as resveratrol (Fig. 4b).

To study the effects of FH1 on NQO2 activity, we used a colorimetric assay that reads out the reduction of a tetrazolium dye, MTT, to its insoluble product formazan, which has a purple color. The NQO2-mediated reduction of menadione to menadiol is coupled to the reduction of MTT by menadiol, resulting in an increase in absorbance at 570nm when active NQO2 is present. We incubated human recombinant NQO2 with menadione, and NMEH as a co-substrate, together with FH1 or other small molecules (i.e. resveratrol, quercetin and imatinib) that have been described to bind and inhibit NQO2 ^29^. We found that FH1 inhibits the activity of NQO2 in a dose-dependent manner (Fig. 4c). Resveratrol had a comparable effect, whereas quercetin and imatinib were more potent NQO2 inhibitors.

Finally, to assess whether NQO2 inhibition itself can have an impact on hepatocyte function in our system, we used siRNA to knock down NQO2 expression in PHHs grown in a monolayer on collagen coated plates. 24 hours after transfection with siRNA, we achieved highly efficient, dose-dependent knockdown of NQO2 expression. Specifically, we detected 82% and 98% reductions in mRNA expression, compared to scrambled control siRNA, using 10nM or 50nM of NQO2 siRNA, respectively. Furthermore, in these conditions, we observed a 1.3- to 1.5-fold elevation in albumin protein secretion (Fig. 4d). This degree of change is comparable to the increase in albumin production elicited by treatment with FH1 (Fig. 1a & Fig. S1), though we cannot exclude the possibility that FH1 has additional targets.

## Discussion

Research using in vitro liver systems is greatly dependent on maintaining the mature functionality of PHHs. By identifying a binding target for the hepatoprotective small molecule FH1, we provide new insight to apply towards efforts to improve the functionality of in vitro liver models.

We showed that the pattern of FH1-induced gene expression includes pathways associated with mature liver function, and a reduced representation of genes that have been implicated in the regulation of fibrosis and liver inflammation. Several transcripts that are upregulated during the acute phase response, i.e. SAA, FGA and FGB, were expressed at significantly lower levels compared to controls, and other positive acute phase reactants followed the same trend. This apparent reduction in inflammatory response pathways is consistent with higher permissiveness to viruses and parasites in this culture setting ^30^. Inflammation, as a tissue state, is a valuable context in which to promote regeneration, or to induce immune responses that lead to the eradication of pathogens, though elevated levels of inflammation and the associated regenerative response are strongly linked with reduced liver function ^31^. Results presented here suggest that in in vitro liver cultures, inhibiting an inflammatory response may concomitantly elevate cultured hepatocyte functionality, at least according to some biosynthesis metrics.

Our current studies demonstrate that FH1 can also enhance the functionality of liver cells in vivo. Using a zebrafish model, FH1 treatment increased embryonic liver sizes during development, and also protected from hepatotoxicity-induced death. Although liver enlargement in adults is generally associated with liver disease and reduced functionality, we did not observe ballooning, cell necrosis, or other markers of hepatic injury after treatment with FH1. On the contrary, we found that FH1 treatment protected both embryos and adult zebrafish against acetaminophen-induced liver toxicity, but only when given in combination with or shortly after acetaminophen administration. As the FH1 molecule resembles two acetaminophen molecules linked together, it could directly compete with the production of NAPQI from acetaminophen. However, a similar critical time window has been observed with other compounds that protect against acetaminophen toxicity, such as gadolinium chloride and N-acetylcysteine. Gadolinium chloride inhibits the activation of Kupffer cells and has been shown to block alterations in NF-κB and IL-6 binding activity, which have been shown to be key players in acetaminophen-induced inflammation and subsequent tissue damage ^32^. Relatedly, N-acetylcysteine replenishes GSH stores and can suppress TNF-induced NF-κB activation through inhibition of IκB kinases ^18,33,34^. It is currently unclear how FH1 treatment leads to liver enlargement, and if the increase in size is driven by an increase of cell size, cell number - via differentiation, proliferation, or protection from death - or a combination. Additional study of the cellular composition of enlarged livers could provide insight into the underlying mechanism behind FH1’s effects.

The growing evidence of a link between FH1, liver function, and reduced inflammation is further strengthened by the identification of NQO2 as a binding partner for FH1. It has been reported that inhibiting NQO2 activity can antagonize the cytotoxic effects of the chemical herbicide paraquat that, just like acetaminophen, can cause oxidative stress and inflammation ^35^. Furthermore, studies using resveratrol have suggested that NQO2 is upstream of and integral to the regulation of NF-κB p65 ^36^. Resveratrol may have additional targets that differ from FH1, e.g. resveratrol can activate the protein deacetylase Sirtuin 1 gene^37^, though expression of the sirtuin family members was not significantly altered upon treatment with FH1. Still, other NQO2 inhibitors were shown to attenuate TNF-mediated, NF-κB-driven transcriptional activity ^38^. Due to the connection between NQO2 and NF-κB signaling, we believe that FH1 exerts its hepatoprotective effects by altering redox-signaling and inhibiting pro-inflammatory cytokines.

While inhibition of pro-inflammatory pathways can result in more stable in vitro cultures of PHHs, following this approach may not be without drawbacks in an in vivo setting. Inflammatory pathways are essential for regeneration after injury. For example, high TNF and NF-κB signaling in fetal hepatocytes and hepatocytes after injury are essential for the (re)generation of liver tissue. For follow up study it would be interesting to analyze the effects of FH1 on regeneration in a partial hepatectomy model.

Even though the identification of NQO2 fits well with the observed data, and NQO2 was the only hit in our affinity pulldown approach that showed a significant difference in enrichment after competitive binding with free FH1, we cannot exclude that FH1 may have additional, likely context-dependent targets. We optimized our bait molecule based on the effectiveness of FH1-analogues to promote cultured PHH cells to secrete albumin. Other mechanisms of action of FH1, involving different targets, may have been lost in this specific work-flow. Furthermore, the mechanism connecting NQO2 to NF-κB signaling is not completely understood, and more research needs to be done to unravel the players downstream of NQO2. Ultimately, this FH1-NQO2 connection could lead to new insight into the relationships between inflammation, regeneration, and tissue functionality, suggest alternative target pathways that promote the functionality of tissues, and inform the engineering of more accurate in vitro systems used to model disease.

## Methods

### Cell culture

Cryopreserved primary human hepatocytes were obtained from Life technologies (lot Hu4175) or from Invitrogen (lot Hu4151, lot Hu8085). Hepatocytes were pelleted by centrifugation at 50g for 10 min and resuspended in hepatocyte medium. 3T3-J2 murine fibroblasts were a gift from Howard Green of Harvard Medical School and were maintained in DMEM with 10% bovine serum (Invitrogen), 10 U/mL penicillin, and 10 mg/mL streptomycin. Cells were cultured in a 5% CO2 humidified incubator at 37°C. Hepatocyte culture medium consisted of Dulbecco’s Modified Eagle Medium (DMEM; Invitrogen) with 10% fetal bovine serum (Invitrogen), 0.5 U/mL insulin (Lilly), 7 ng/mL glucagon (Bedford Laboratories), 7.5 μg/mL hydrocortisone (Sigma), 10 U/mL penicillin (Invitrogen), and 10 mg/mL streptomycin (Invitrogen).

### Screening

Analogue screens to determine structure activity relationship were performed according to Shan et al., ^12^. In short, 384-well glass bottom plates (Corning) were coated with type I collagen in water (100 μg/ml; BD Biosciences) for 1 h at 37 °C. A feeder layer of J2–3T3 fibroblasts was plated onto the collagen at a density of 8,000 cells per well and allowed to reach confluence over 48 h. Then, primary human hepatocytes were seeded onto the fibroblasts at a density of 2,000 cells per well and maintained under standard culture conditions with daily replacement of the hepatocyte medium for 7 d. Compounds were added in triplicate on day 7 for 48 h. On day 9, culture supernatants were analyzed by competitive albumin ELISA (MP Biomedicals) using horseradish peroxidase detection and chemiluminescent luminol (Pierce) as a substrate.

### RNAseq analysis

3T3-J2 cells were seeded in a collagen coated 12-well plate (500,000 per well) and allowed to reach confluency. 135,000 primary human hepatocytes (Hu4175, Hu4151 or Hu8085) were seeded on top of the 3T3-J2 feeder layer and small molecules were added 24h after seeding. Each donor was treated with DMSO, or FH1 in a final concentration of 20μM, 5μM, or 1.25μM. After 24h of treatment, cells were lysed and RNA was isolated and analyzed by RNA sequencing. We determined dose responses for hepatocytes from three different donors; each donor represents one biological replicate in the rest of the analysis. Extended methods explaining the technical RNAseq analysis can be found in the supplemental information. Functional profiling was done using g:Profiler on the top 100 genes sorted for fold change between 20μM FH1 and the DMSO control.

### Zebrafish experiments

Zebrafish were maintained according to Harvard Medical School Institutional Animal Care and Use Committee protocol. Zebrafish were exposed to FH1 dissolved in DMSO and/or acetaminophen dissolved in water. Whole-mount in situ hybridization; paraformaldehyde-fixed animals were processed for in situ hybridization according to standard zebrafish protocols (www.zfin.org), using probes for fabp10a. Animals were imaged using a Zeiss Discovery V8/AxioCam MRc with the Axiovision software suite. Liver size (fabp10a expression) was examined in a blinded fashion following in situ hybridization and larvae were classified as having a “small”, “normal”, or “large” liver.

### Linker addition

The following chemicals (Sigma) were used to perform the coupling reaction depicted in Fig. 3c: HOBT = Hydroxybenzotriazole, EDCL = N-(3-Dimethylaminopropyl)-N′-ethylcarbodiimide hydrochloride, DIPEA = N,N-Diisopropylethylamine, DMF = Dimethylformamide. Boc (t-butyl carbamate) deprotection was done using TFA/CH2CL2 (Trifluoroacetic acid).

### Affinity pulldown using magnetic beads

For each condition, 0.1 mL of Pierce NHS-Activated Magnetic Beads solution (Thermofisher) was aliquoted in 1.5ml Eppendorf tubes, washed 4 times with anhydrous DMSO and adjusted to 50% slurry with anhydrous DMSO (~0.2 mL total). 24 μL of 10 mM FH1-analogue compound solution was added, or 24 uL of DMSO for the empty beads control. 1.5 μL of triethylamine was added to each tube and incubated overnight at room temperature with rotation, protected from light. The second day, 5 μL aminoethanol was added to each tube and incubated overnight to block the beads. The third day, beads were washed 3x with 0.5 mL anhydrous DMSO, and 3x with lysis buffer (100 mM MES, 1 mM EGTA, 0.5 mM MgCl_2_ [pH 6.7]) containing 1 mM PMSF and 0.1% NP-40). After washing, beads were incubated with ~ 1 mg of total protein lysate from donor Hu4175, overnight at 4 degrees Celcius. For the competitive binding control, lysates were pre-incubated with 100 μM FH1 small molecule for three hours prior to the addition of bait-beads. The fourth day, beads were washed 3x with PBS and bound proteins were eluted with 0.1mL 0.1M glycine-HCl, pH 2.5–3.0, and neutralized with 1M Tris-HCl, pH 8.5 before submitting samples for quantitative proteomics. We used isobaric tags for relative and absolute quantitation (iTRAQ) in combination with tandem mass spectrometry to compare the samples.

### Molecular docking analysis

Docking analysis was done using the web-based interface www.dockingserver.com ^27^. The structure of FH1 was manually added, the structure of resveratrol was pulled from the PubChem data bank. The confirmation of the active site of NQO2 was pulled from the RCSB protein data bank, more specifically 1SG0-oxidoreductase. Docking was done using default parameters; torsion (0.2 Ã), rigid-body orientation (5Â°), dihedral angles (5Â°), root mean square deviation tolerance (2.0 Ã).

### Cellular Thermal Shift Assay (CETSA)

CETSA was performed as in ref ^39^. In brief, HPPs grown on collagen-coated plates were treated with compounds in DMSO (final concentration not exceeding 0.1% (w/v)) for 24h. Cells were trypsinized, washed with PBS, and suspended in PBS supplemented with protease inhibitor cocktail (Sigma-Aldrich). The cell suspensions (5000 cells/μL) were then heated at the indicated temperature for 3 min and lysed with three freeze–thaw cycles using dry ice and a 42 °C water bath. Cell lysates were centrifuged at 16000g for 15 min at 4 °C, and analyzed by western blot, blotting for NQO2 (nbp1–31563), and β-Tubulin (ab6046) as a loading control.

### NQO2 activity assay

Inhibition of NQO2 activity was measured by mixing 50 μL assay buffer ( 50 mM Hepes-KOH, pH 7.4 with 0.01% Tween20, 0.18 mg/mL BSA, and 1 μM FAD) containing 100 ng of recombinant NQO2 (Sigma-Aldrich), with 50 μL of the same buffer with test compounds or DMSO (control). Reactions were initiated by adding 50 μL of assay buffer containing 500 μM NMEH as co-substrate as well as 600 μM MTT and 300 μM menadione. After 10 minutes, the absorbance of the samples was measured at 595 nm in a 96-well assay format. Background measurements were made using a sample without recombinant NQO2.

### siRNA knockdown

Knockdown of NQO2 was done using SMARTpool: ON-TARGETplus siRNA (Dharmacon) in combination with Lipofectamine RNAiMax (Thermofisher) according to manufacturers instructions. 10,000 primary human hepatocytes were seeded per collagen-coated 96-well well, and transfected after 24h with the designated final concentration of siRNA. 48h after siRNA transfection, supernatants were collected and analyzed by albumin ELISA.

## Supplementary Material

supplement

## Figures and Tables

**Table 1.** Down-regulated genes upon FH1 treatment

Gene	GeneID	Fold change	p-value
ENSG00000166741	NNMT	0.73	0.001
ENSG00000181830	SLC35C1	0.73	0.001
ENSG00000134339	SAA2	0.75	0.003
ENSG00000159674	SPON2	0.78	0.007
ENSG00000188157	AGRN	0.80	0.016
ENSG00000171560	FGA	0.84	0.025
ENSG00000205358	MT1H	0.81	0.025
ENSG00000112096	SOD2	0.82	0.027
ENSG00000124942	AHNAK	0.83	0.028
ENSG00000139269	INHBE	0.81	0.030
ENSG00000118804	FAM47E-STBD1	0.82	0.033
ENSG00000115457	IGFBP2	0.82	0.035
ENSG00000203832	NBPF20	0.82	0.035
ENSG00000197728	RPS26	0.85	0.042
ENSG00000177606	JUN	0.82	0.045
