# Supplementary material for: Identification of NQO2 as a protein target in small molecule modulation of hepatocellular function
Source: ACS Chem Biol. Author manuscript; Available in PMC 2026 Mar 9. (PMC12969845; doi:10.1021/acschembio.1c00503)

Fig.S1 Effect of FH1 on hepatocytes with and without 3T3-J2s

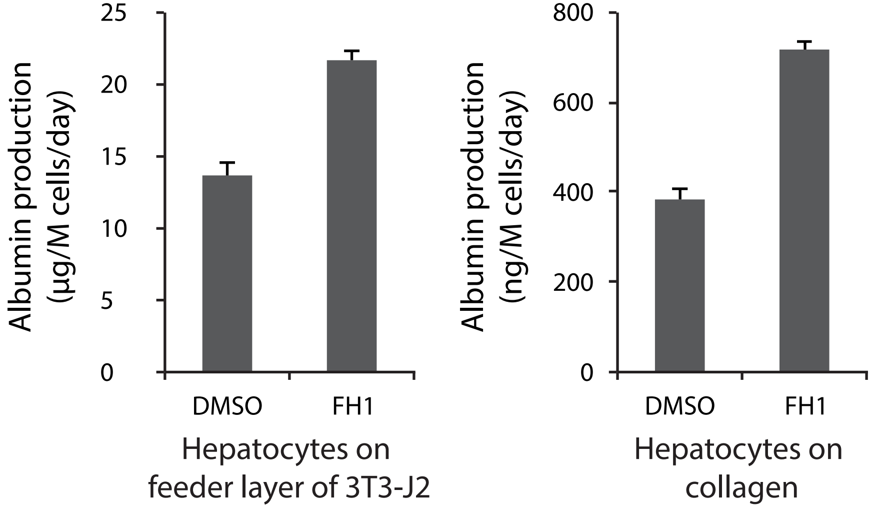

Fig.S2 Effects of FH1 on liver size in vivo

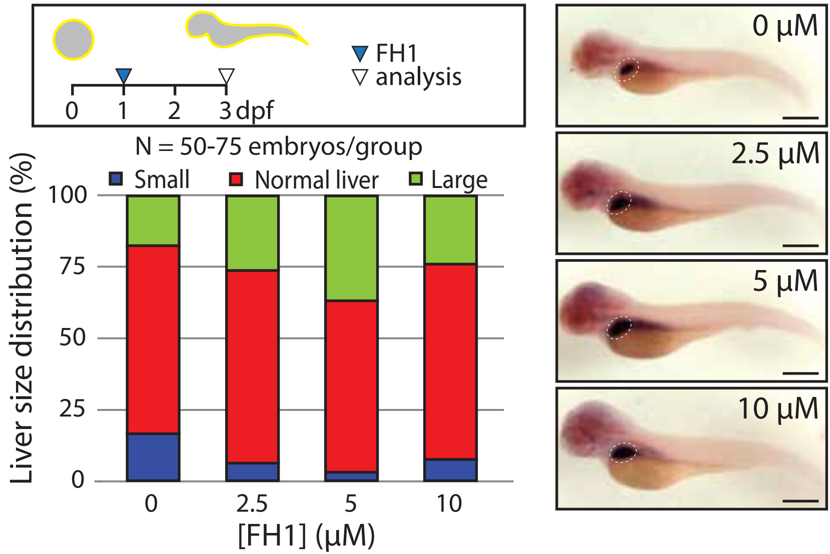

Quantification of liver size in treated and control groups.
One day old zebrafish embryos were treated with different doses of FH1 for two days, and liver size was examined by whole-mount in situ hybridization for fabp10a, which is specifically expressed in hepatocytes (dpf = days post fertilization). Following WISH, larvae were classified in a blinded fashion as having a "small", "normal", or "large" liver. Representative images of whole-mount in situ hybridization for fabp10, after treatment with varying doses of FH1 illustrate the increase in liver size. Scale bars represent 500 µm.

Fig.S3 Custom FH1 analogues

A series: 18 targets

R = H; CH_3; CH_2CH_3; NH_2; OH; OMe

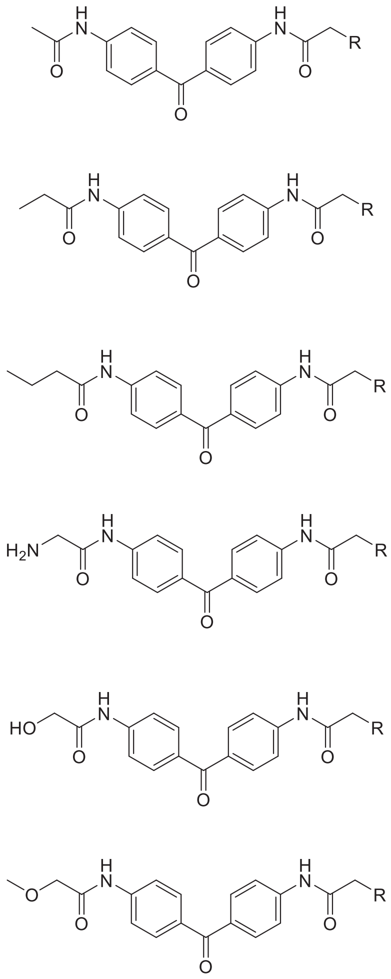

B series: 18 targets

R = H; CH_3; CH_2CH_3; NH_2; OH; OMe

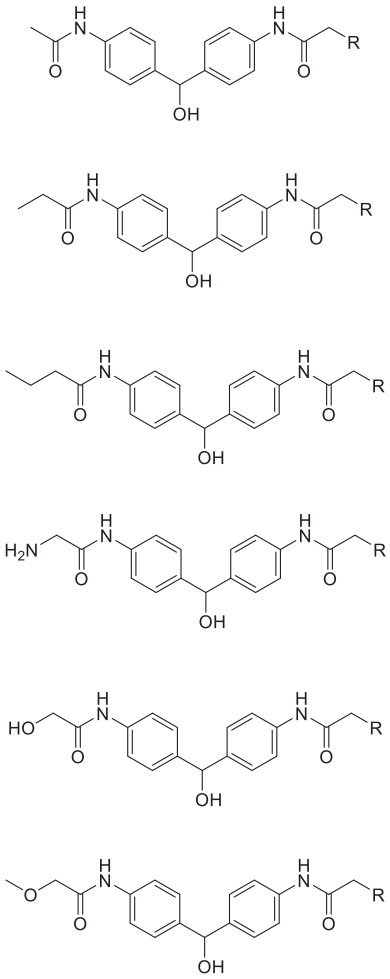

C series: 18 targets

R = H; CH_3; CH_2CH_3; NH_2; OH; OMe

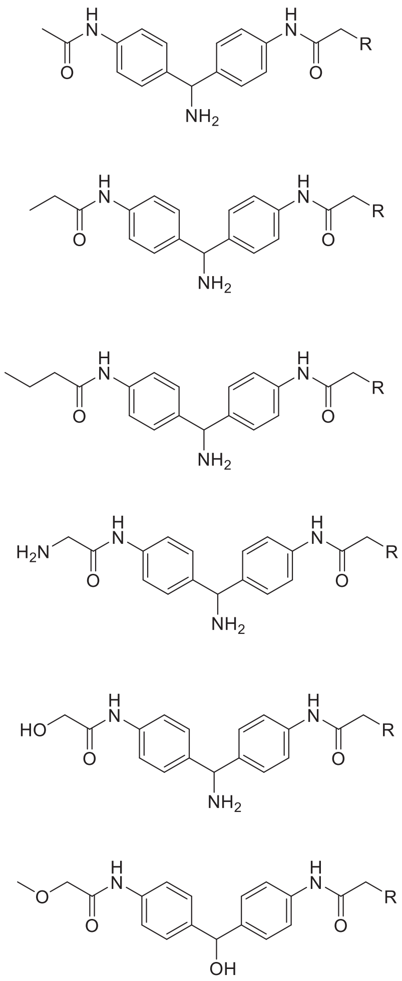

D series: 18 targets

R = H; CH_3; CH_2CH_3; NH_2; OH; OMe

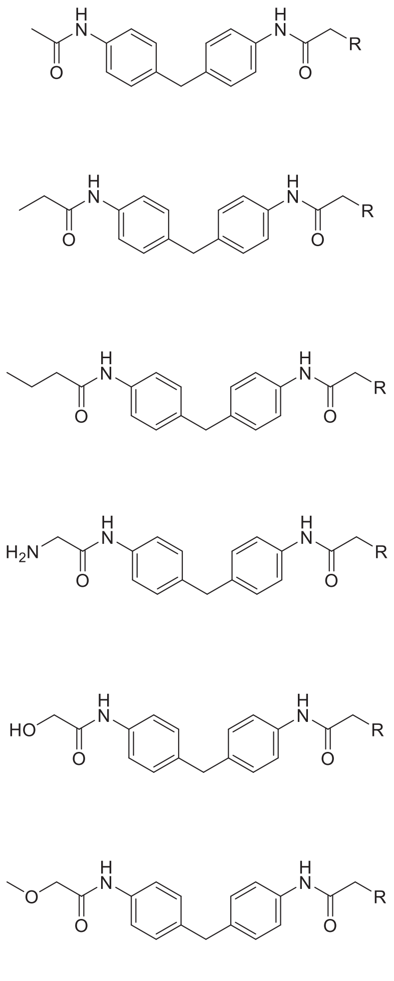

Fig.S4 Affinity pulldown targets

	Peptide counts		Relative protein abundance						Average protein abundance			Enrichment
Gene name	Total	Unique	DMSO	DMSO	Bait	Bait	comp. binding	comp. binding	DMSO	Bait	Comp. binding	Bait vs comp. binding
UQCRH	10	2	1.0	1.2	8.4	7.0	6.7	8.2	1.1	7.7	7.4	1.04
ATP5J	17	5	1.0	1.1	5.5	4.8	4.8	4.1	1.0	5.1	4.5	1.15
ATP5D	22	3	1.0	1.1	4.2	3.6	3.3	3.0	1.0	3.9	3.1	1.24
**NQO2**	2	2	1.0	1.1	3.9	3.7	1.5	1.2	1.0	3.8	1.4	2.77
GIN1	3	1	1.0	1.0	3.6	2.7	2.5	1.9	1.0	3.2	2.2	1.42
ECHS1	36	13	1.0	1.1	3.4	2.7	2.8	2.7	1.0	3.1	2.7	1.12
HSPA8	19	8	1.0	1.1	2.6	2.2	2.1	2.0	1.0	2.4	2.1	1.15
NDUFA2	1	1	1.0	1.1	2.2	2.1	2.0	2.2	1.1	2.1	2.1	1.02
RPSA;GALP;ZFYVE26	4	1	1.0	1.0	2.1	2.0	2.0	1.7	1.0	2.0	1.8	1.11
HSPA5	78	25	1.0	1.1	2.0	1.9	1.8	1.6	1.0	2.0	1.7	1.13
HSPA6; HSPA2; HSPA8 ; HSPA1B	2	1	1.0	1.0	2.0	1.8	1.7	1.4	1.0	1.9	1.5	1.23
HSPA2; HSPA8; HSPA5; HSPA1L	3	1	1.0	1.1	2.0	1.8	1.9	1.6	1.1	1.9	1.7	1.08
TRAP1	1	1	1.0	1.1	2.1	1.7	1.6	1.8	1.0	1.9	1.7	1.09
CLTC; CLTCL1	29	13	1.0	1.1	2.0	1.7	1.7	1.5	1.1	1.8	1.6	1.14
CALU	1	1	1.0	1.2	1.9	1.7	1.8	2.0	1.1	1.8	1.9	0.95
HSPD1	25	16	1.0	1.0	1.9	1.7	1.7	1.7	1.0	1.8	1.7	1.05
PDZD11; USP25	1	1	1.0	1.1	1.8	1.7	1.6	1.6	1.0	1.7	1.6	1.09
ATP5B	19	12	1.0	1.0	1.8	1.7	1.8	1.5	1.0	1.7	1.6	1.07
HSPA2; HSPA8	6	5	1.0	1.1	1.7	1.6	1.5	1.2	1.1	1.6	1.3	1.21
CHCHD4	2	1	1.0	0.9	1.7	1.5	1.5	1.7	1.0	1.6	1.6	0.99
HSPA2; HSPA8; HSPA5;HSPA1B	12	2	1.0	1.1	1.7	1.5	1.4	1.3	1.0	1.6	1.3	1.21
TCEB2	1	1	1.0	1.2	1.6	1.5	1.4	1.3	1.1	1.6	1.3	1.16
NPM1	23	5	1.0	1.3	1.7	1.4	1.3	1.4	1.2	1.5	1.4	1.13
DNAJB11	5	3	1.0	1.1	1.5	1.5	1.6	1.2	1.1	1.5	1.4	1.09
YWHAG	2	2	1.0	0.9	1.3	1.8	1.8	1.2	1.0	1.5	1.5	1.01
LPA	2	1	1.0	1.3	1.6	1.5	1.2	1.0	1.2	1.5	1.1	1.36
ATP5EP2, ATP5E	6	2	1.0	1.1	1.4	1.6	1.3	1.0	1.1	1.5	1.1	1.32
EFHD1; EFHD2	3	1	1.0	1.6	1.6	1.4	1.6	1.3	1.3	1.5	1.5	1.02
EFHD1	13	5	1.0	1.4	1.6	1.4	1.6	1.3	1.2	1.5	1.5	1.01
HSPA9	41	15	1.0	1.1	1.6	1.4	1.5	1.2	1.0	1.5	1.4	1.10
CD59	1	1	1.0	0.9	1.6	1.4	1.0	1.3	1.0	1.5	1.1	1.33

Fig.S5 Resveratrol binding to NQO2

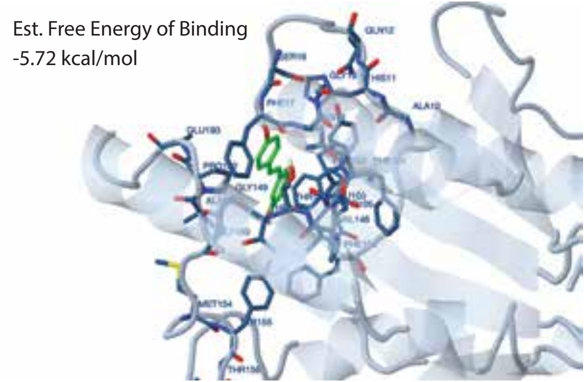

Supplement: supplement [file NIHMS2099766-supplement-supplement.pdf]
